# Supplementary material for: Anti-dsDNA B-Cell ELISpot as a Monitoring and Flare Prediction Tool in SLE Patients
Source: J Clin Med. 2023 Feb 6;12(4):1295. doi: 10.3390/jcm12041295 (PMC9958982; doi:10.3390/jcm12041295)
Supplement: Supplementary file 1 [file jcm-12-01295-s001.zip › jcm-2171862-supplementary.pdf]

Supplementary Table S1. Different conditions tested from the original ones in Hanaoka design. In bold, conditions selected for the ELISpot.

	Hanaoka conditions	Conditions tested
Samples	N/A	Fresh, frozen
DNA coating	Albumin	Albumin, DNA Coating Solution
PBMCs	500,000 cells/well	0.5M cells/well, 1M cells/well, 2M cells/well, 4M cells/well, 8M cells/well
Replicate wells	5 wells	3 wells, 5 wells, 7 wells
DNA origin	Lambda phage	Beef thymus, lambda phage
Nuclease treatment	Treated DNA	Treated DNA, untreated DNA
DNA concentration	100 μg/mL	200 μg/mL, 100 μg/mL
Blocking solution	N/A	100 μL, 150 μL

Abbreviations: M (million).
